# Thermosensitive hydrogels deliver bioactive protein to the vaginal wall

**DOI:** 10.1371/journal.pone.0186268

**Published:** 2017-10-26

**Authors:** Meadow M. Good, T. Ignacio Montoya, Haolin Shi, Jun Zhou, YiHui Huang, Liping Tang, Jesus F. Acevedo, R. Ann Word

**Affiliations:** 1 Division of Female Pelvic Medicine and Reconstructive Surgery, Department of Obstetrics and Gynecology, University of Texas Southwestern Medical Center, Dallas, TX, United States of America; 2 Department of Bioengineering, University of Texas at Arlington, Arlington, TX, United States of America; University of South Carolina, UNITED STATES

## Abstract

The pathophysiology and natural history of pelvic organ prolapse (POP) are poorly understood. Consequently, our approaches to treatment of POP are limited. Alterations in the extracellular matrix components of pelvic support ligaments and vaginal tissue, including collagen and elastin, have been associated with the development of POP in animals and women. Prior studies have shown the protease MMP-9, a key player of ECM degradation, is upregulated in vaginal tissues from both mice and women with POP. On the other hand, fibulin-5, an elastogenic organizer, has been found to inhibit MMP-9 in the vaginal wall. Hence, we hypothesized that prolonged release of fibulin-5 may delay progression of POP. To test the hypothesis, oligo (ethylene glycol)-based thermosensitive hydrogels were fabricated, characterized and then used to deliver fibulin-5 to the vaginal wall and inhibit MMP-9 activity. The results indicate that hydrogels are cell and tissue compatible. The hydrogels also prolong the ½ life of fibulin-5 in cultured vaginal fibroblasts and in the vaginal wall in vivo. Finally, fibulin-5-containing hydrogels resulted in incorporation of fibulin-5 into the vaginal matrix and inhibition of MMP-9 for several weeks after injection. These results support the idea of fibulin-5 releasing hydrogel being developed as a new treatment for POP.

## Introduction

It has been estimated that 11% of women will undergo at least one surgical procedure for vaginal prolapse or incontinence during their lifetime [1, 2]. Due to different classification systems, unclear reports for symptomatic vs asymptomatic women, and the unknown lack of medical attention for prolapse, it is difficult to ascertain the exact prevalence of pelvic organ prolapse (POP). Nonetheless, interviews and questionnaires indicate that prolapse is common (varying between 15–48% after childbirth [3]) and results in significant impairment of quality of life. Despite its common prevalence, the pathophysiology and natural history of pelvic organ prolapse (POP) are poorly understood. Given our minimal understanding of the underlying mechanisms, our approaches to treatment of POP are limited, with reported surgical outcome failure rates as high as 56% over 6–10 years and up to one third of annual POP surgeries representing repeat procedures [2, 4].

Support of the vaginal wall is provided by elastic fibers in the extracellular matrix, facilitating elasticity and recoil to tissues, which results in structural integrity against mechanical strain. Elastic fibers consist of an elastin core surrounded by proteins, including fibulin-5, a key protein that not only promotes elastogenesis but also inhibits matrix metalloproteinase-9 (MMP9), an enzyme that degrades extracellular matrix proteins, including collagen and elastin in the vaginal wall [5–7]. Fibulin-5 binds cell surface integrin receptors that extend from the cell surface into the extracellular matrix, activating signaling pathways that suppress MMP9. Interestingly, the integrin binding domain of fibulin-5 is crucial for suppression of MMP9 but dispensable for elastogenesis [6]. Therefore, fibulin-5 plays two critical roles in elastic fiber homeostasis in the extracellular matrix: facilitating the formation of elastic fibers and inhibiting matrix degradation.

Alterations in collagen, elastin and proteoglycan proteins of the extracellular matrix within the pelvic support ligaments and vaginal tissue have been associated with the development of POP in animals and in women [5]. Elastic fiber homeostasis is crucial in maintaining pelvic floor support; without this careful balance, connective tissues deficient fibulin-5 expression in mice [8, 9] and women develop POP [6]. Moreover, MMP9 is increased in connective tissues of the pelvic floor in mice and women with pelvic organ prolapse [6, 10, 11].

Graft materials in pelvic organ prolapse and abdominal hernia repair have shown promise. However, their successful incorporation into native tissues has been difficult to achieve. Restoring the natural extracellular matrix microenvironment in the abdominal and vaginal walls is complex and requires key proteins that promote collagen and elastin synthesis. Combining synthetic and natural biomaterials may improve cell survival, provide scaffolds for cell regeneration, and enhance connective tissue remodeling.

Introduction of a biologically active protein into tissue presents opportunity to rescue protein degradation. An optimal carrier material should be able to stabilize and deliver protein slowly into the surrounding tissue. Injectable hydrogel scaffolds have been developed for sustained protein release without the use of solvents which are commonly used for polymeric scaffold fabrication and may denature or inactivate biologically active proteins [12–14]. Hence, some research efforts have been placed on developing in-situ gelling hydrogels by chemical crosslinking for delivery of protein drugs [15, 16]. For example, Prestwich etc. developed an injectable, in-situ forming hyaluronan hydrogel for supporting cell proliferation and growth to permit in vivo engineering of new tissues [17]. Although the system shows some excellent properties such as cell-benign environment, fast gelling and tailorable mechanical strength, the *in vivo* applications of these hydrogels may have many limitations, which include potential cytotoxicity of the chemical crosslinkers or reaction byproducts. To overcome these shortcomings, many thermally-induced gelling systems have been developed in recent years [15, 16]. These injectable hydrogel scaffolds are based on an inverse thermo-reversible gelation effect, in which the carrier is liquid at room temperature (allowing the protein to be easily mixed and subsequently injected) but gels with no requirement of toxic crosslink agents at body temperature trapping the protein with subsequent slow release. Here, we tested the hypothesis that delivery of purified recombinant fibulin-5 protein to the vaginal wall using a nontoxic, non-immunogenic oligo (ethylene glycol)-based thermogelling hydrogel (HG) would inhibit MMP-9 in the vaginal wall of *Fbln5* knockout mice. The results of this work would provide evidence to support the hypothesis that slow release of fibulin-5 could delay the progressions of POP.

## Materials and methods

### Materials

Polymers and chemicals, including 2-(2-Methoxyethoxy) ethyl methacrylate (MEO_2_MA), oligo(ethylene glycol) monomethyl ether methacrylates (OEOMA, M_w_:475), acrylic acid (AAc) and 4,4'-Azobis(4-cyanopentanoic acid) (ACPA), were purchased from Aldrich. All animals were handled and euthanized in accordance with the standards of humane animal care described by the National Institutes of Health Guide for the Care and Use of Laboratory Animals, using protocols approved by the Institutional Animal Care and Use Committee (IACUC) of the University of Texas Southwestern Medical Center in Dallas, Texas. A total of 71 *Fbln5*^*-/-*^ KO mice were housed in IACUC approved facilities under a 12-hour light cycle at 22°C.

### Fabrication of thermogelling polymer

Themogelling hydrogel polymer based on oligo (ethylene glycol) macromonomer was synthesized as described previously with minor modification[18]. Briefly, MEO_2_MA (0.96M), OEOMA (0.04M), AAc (0.028M) and ACPA (0.5mM) as an initiator were dissolved in 100 ml of ethanol in a reactor. The solution was purged with nitrogen gas for 10 min at room temperature, the reactor was then placed in a water bath at 68°C and polymerization proceeded for 6 h. These crude polymers were collected via removal of ethanol with evaporation under vacuum and the crude polymer was then re-dissolved in DI water, purified with dialysis and lyophilized before use.

### Characterization of thermogelling polymer

#### Molecular weight

Molecular weight and polydispersity index of the polymer was measured by gel permeation chromatography (Viscotech GPCmax, PLgel 5μm MIXED-D columns by Polymer Labs). Tetrahydrofuran was used as eluent and eluting flow rate was set at 1.0 mL/min.

#### Temperature-dependent phase transitions

The phase transition temperature of the polymer was determined based on temperature dependent light transmittance using a UV-vis spectrometer [18]. Briefly, 1.5 mL of polymer aqueous solution (0.5 wt%) was added into a 1-cm UV cuvette covered with a lid to prevent water evaporation during the test. The cuvette was then placed into a water bath with a temperature controller. The temperature was manually increased at an interval of 1°C. After the temperature reached the designated degree, the sample was further incubated for 10 minutes before measuring transmission of light at 500 nm.

#### In vitro cell toxicity

Briefly, 1 ml hydrogel (25 wt%) was incubated with 1 ml of DMEM media for different periods of times (1, 3, 5 and 7 days). The supernatants were collected and then cultured with 3T3 fibroblasts (ATCC, Manassas, VA) for 24 days. The cell numbers of various groups were then quantified using MTT assays as described earlier [19].

#### In vivo biocompatibility

A mouse subcutaneous implantation model was used to determine in vivo biocompatilibity of the polymer [20, 21]. Briefly, 8 week-old BALB/c mice (male, 20–25 g body weight; Taconic Farms, Germantown, New York) were implanted subcutaneously on the back with 0.1 mL of the hydrogel (100mg/ml). After implantation for one week, the mice were sacrificed. The implants as well as surrounding tissues were isolated, frozen sectioned and stained with hematoxylin-eosin. The numbers of cells surrounding implants were quantified to reflect the extent of foreign body reactions.

#### Protein release studies

Animal studies were carried out to assess the slow release property of a model protein, bovine serum albumin (BSA), from the hydrogel as described earlier [20]. Briefly, BSA was conjugated with near-infrared (NIR) dye (Oyster®-800, Boca Scientific) by following manufacturer’s instructions and NIR-labeled BSA was then injected into the vaginal wall of mice with or without the presence of hydrogel (100μl, 0.1mg/ml BSA, 25 wt % HG). Release of NIR-BSA was then monitored daily using Kodak In Vivo FX Pro system. For image analyses, regions of interest were drawn over the injection sites in the fluorescence images, and mean intensities for all fluorescent pixels were calculated. These animal experiments and care were approved by Animal Care and Use Committee at the University of Texas at Arlington.

### Recombinant fibulin-5

Recombinant fibulin-5 was purified using mammalian expression system. The expression vector was constructed and utilized in previous experiments by collaborators [22]. Purified protein was collected from conditioned medium from the CHO cell cultures (ATCC, Manassas, VA) using immobilized metal affinity chromatography (IMAC) followed by desalting column chromatography as previously reported [8]. We routinely prepared ~5 L of conditioned media for ~1 mg of recombinant protein. Purity (> 95%) was assessed by silver staining.

### Cell culture

Vaginal stromal cultures from WT and Fbln5^-/-^ mice were generated as previously described [6].

### Fbln5 knockout mice study

The posterior vaginal wall of young (2–4 months of age, n = 33) *Fbln5*^*-/-*^ KO mice were injected with 100 μL of HG alone (25 wt% in water) or with purified recombinant fibulin-5 (HG+fibulin-5, 5–10 μg/ml) using a 23 gauge needle. Animals were anesthetized by isoflurane and monitored every 15 min x 1 h, then every day for any adverse reactions including loss of appetite, vaginal discharge, frequent grooming of the perineal area, poor coat consistency, or frequent respirations. Vaginal tissue was harvested after 1, 2, and 4 wks after euthanasia with isoflurane and exsanguination. Fine microsurgical instruments and a dissection microscope were used to open the abdominal cavity, disarticulate the pubic symphysis, and dissect the uterine horns, bladder, cervix and vagina to the perineal skin. The perineal skin was removed and the vaginal tube was opened. The posterior vaginal wall was isolated and this tissue was either fixed for histologic analysis, or, after scraping off the epithelium, frozen in liquid N_2_ and stored at -80°C for further studies.

### Gelatin zymography

Protein samples (10 ug/lane) were applied to gelatin polyacrylamide minigels (Invitrogen, Carlsbad, CA) (10%) in standard SDS loading buffer containing 0.1% SDS and separated by electrophoresis at room temperature using 125 V. After electrophoresis, the gels were soaked with gentle shaking in renaturing buffer [2.7% (vol/vol) Triton X-100 in distilled water] in a shaker for 30 min with one exchange to new buffer at 15 minutes. Next, the gels were soaked with gentle shaking in assay buffer (50 mmol/l Tris, 200 mmol/l NaCl, 10 mmol/l CaCl2, 0.05% Brij 35, pH 7.5) overnight at 37°C and then stained with Coomassie brilliant blue-R 250 in 50% methanol and 10% acetic acid. The gel was imaged and areas of lysis quantified using the Fuji LAS 3000 image analysis system.

### Immunoblot assays

Protein samples (10 ug/lane) were applied to 20% gradient polyacrylamide gels (Bio-Rad, Hercules, CA), separated by electrophoresis and transferred to nitrocellulose membranes overnight at 4°C. Identical gels were run side-by-side and Coomassie blue-stained for protein loading comparison among samples. After protein transfer, membranes were treated with blocking buffer (TBS-T with 2.5% non-fat milk) for 1 hour. The membranes were then treated with primary antibody (His-tag, 1:1000 and BSYN-1923, 1:250) for 60 minutes at 37°C. Membranes were then serially washed with TBS-T, followed by treatment with the secondary antibody (goat IgG anti-rabbit, 1:2000) at room temperature.

### Hydroxyproline assay

Collagen solubility measurements were used to ascertain collagen content within the vaginal tissue [23]. Weights of vaginal tissues were determined before and after lyophilization. Lyophilized tissue was homogenized with 1M NaOH with protease inhibitors (PIs) (16 mM potassium phosphate, pH 7.8, 0.12 M NaCl, 1 mM ethylenediaminetetraacetic acid, 0.1 mM phenylmethylsulfonyl fluoride, 10 μg/ml pepstatin A, and 10 μg/ml leupeptin) at 4°C for 24 hrs, centrifuged and the supernatant removed (newly synthesized, non-cross linked collagen fraction). The remaining residue was washed with water and PI and extracted with 0.5M acetic acid + PI at 4oC for 24 h with rotation. Samples were centrifuged and the supernatant saved as Fraction B (denatured mature collagen). The remaining tissue pellet was saved as Fraction C (mature, cross-linked collagen). Collagen content in each fraction was determined by measurement of hydroxyproline content by the chloramine (13)-T method after overnight hydrolysis in 6M HCl at 100°C. Hydroxyproline values were converted to collagen (x 7.6 constant) and normalized to tissue wet weight.

### Histology

Rings from cross-sections of injected vaginal wall were fixed in 10% formalin x 24 h, then changed to 80% ethanol. The vaginal wall was oriented in the cassette which was then paraffin embedded. Sections (5 μm) were stained with Masson’s trichrome staining. Thereafter, thickness of vaginal epithelium and stroma was quantified using the average of 4–5 measurements from each compartment.

### Statistical analysis

Data were analyzed using ANOVA with Dunnett’s test assigning a control group. Statistical analysis was accomplished using SAS, version 9.2 (SAS institute, Cary, N.C.).

## Results and discussion

### Characterization of thermogelling polymer for delivery of bioactive agents

**Fig 1:**
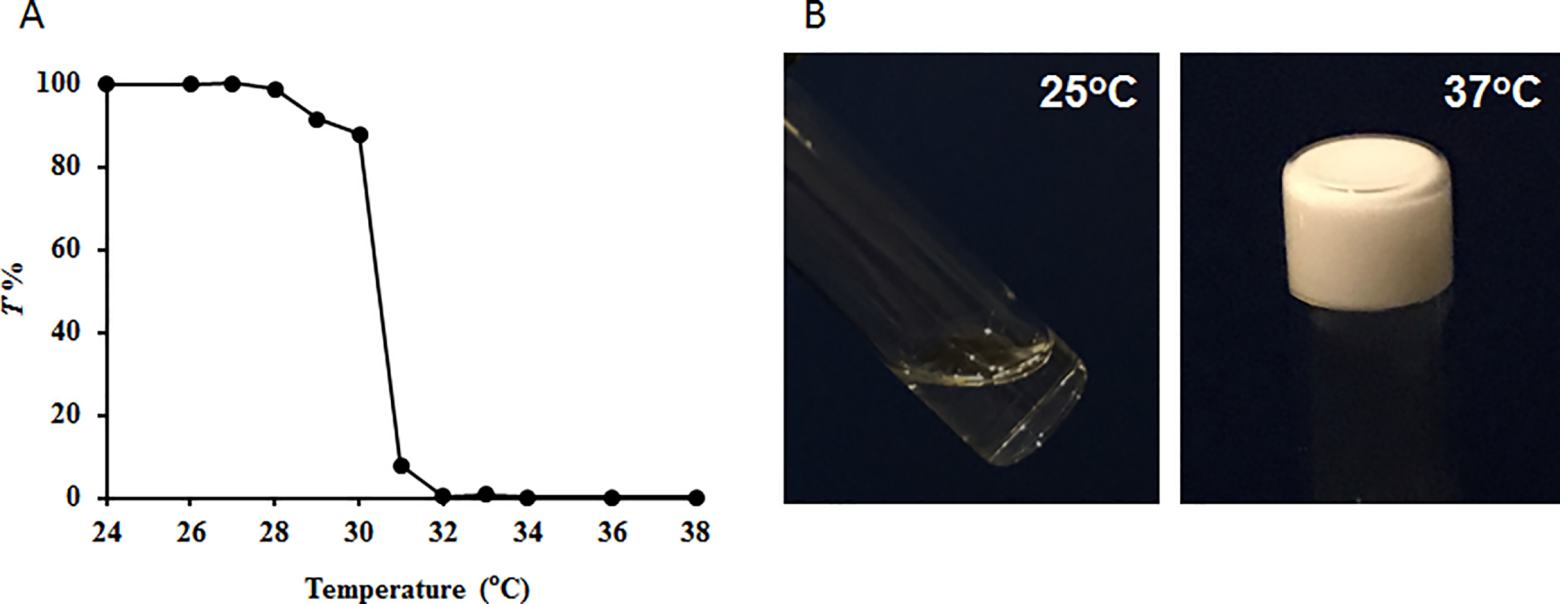
Thermosensitive property of the polymer solution. **A**. Transmittance of the polymer aqueous solution (0.5 wt%) as a function of temperature. **B**. Thermally-induced gelation of the HG aqueous solution (25 wt%).

To overcome drawbacks of solvents in hydrophobic polymers that denature and/or deactivate proteins [13, 14], many hydrophilic thermogelling hydrogels have been developed. Typically, these carriers are based on an inverse thermoreversible gelation effect, in which the carrier is in liquid form at room temperature, allowing the protein to be easily mixed and subsequently injected into the site of interest [24]. At body temperature, the carrier material forms a gel, retarding protein diffusion rate and resulting in slow release of proteins. Among all injectable hydrogels, many of them contain copolymers of poly-N-isopropylacrylamide-co-acrylic acid (PNIPAM) [25, 26]. Despite good protein slow release properties, many concerns have been raised regarding the long-term safety of PNIPAM. Specifically, the hydrolysis of PNIPAM-based polymers may produce derivatives that have neural and reproductive toxicity [27, 28]. To circumvent this problem, we had developed a oligo(ethylene glycol)-based thermogelling hydrogel [20]. It is well established that PEG is nontoxic and non-immunogenic [29, 30]. Here a thermogelling polymer (HG) was synthesized via free radical polymerization. GPC measurement showed a number average molecular weight (M_n_) of 34760 and a PDI of 2.78. Phase transition temperature of the HG was determined using a UV-Visible spectrometer in which visible light transmittance at 500 nm over temperature for the diluted HG polymer aqueous solution (0.5 wt%) was measured (Fig 1A). The results indicate that transmittance changed slightly between 26 to 30°C (Fig 1A). However, the transmittance dropped sharply between 30 and 31°C, and remained steady thereafter. Phase transition temperature was estimated to be at 30.5°C. Similar results had already been reported for PNIPAM-co-AAc polymer [31]. Further, the HG aqueous solution (25 wt%), showed transparency, and even viscous (kinematic viscosity:58.5 mm^2^/s at 20°C, determined by a Cannon^TM^ Ubbelohde viscometer, Fisher Scientific), could still flow at room temperature. However, it became opaque and stopped flowing to form hydrogel as observed in an inverted vial (in ~ 7 seconds) when heated to 37°C (Fig 1B).

**Fig 2:**
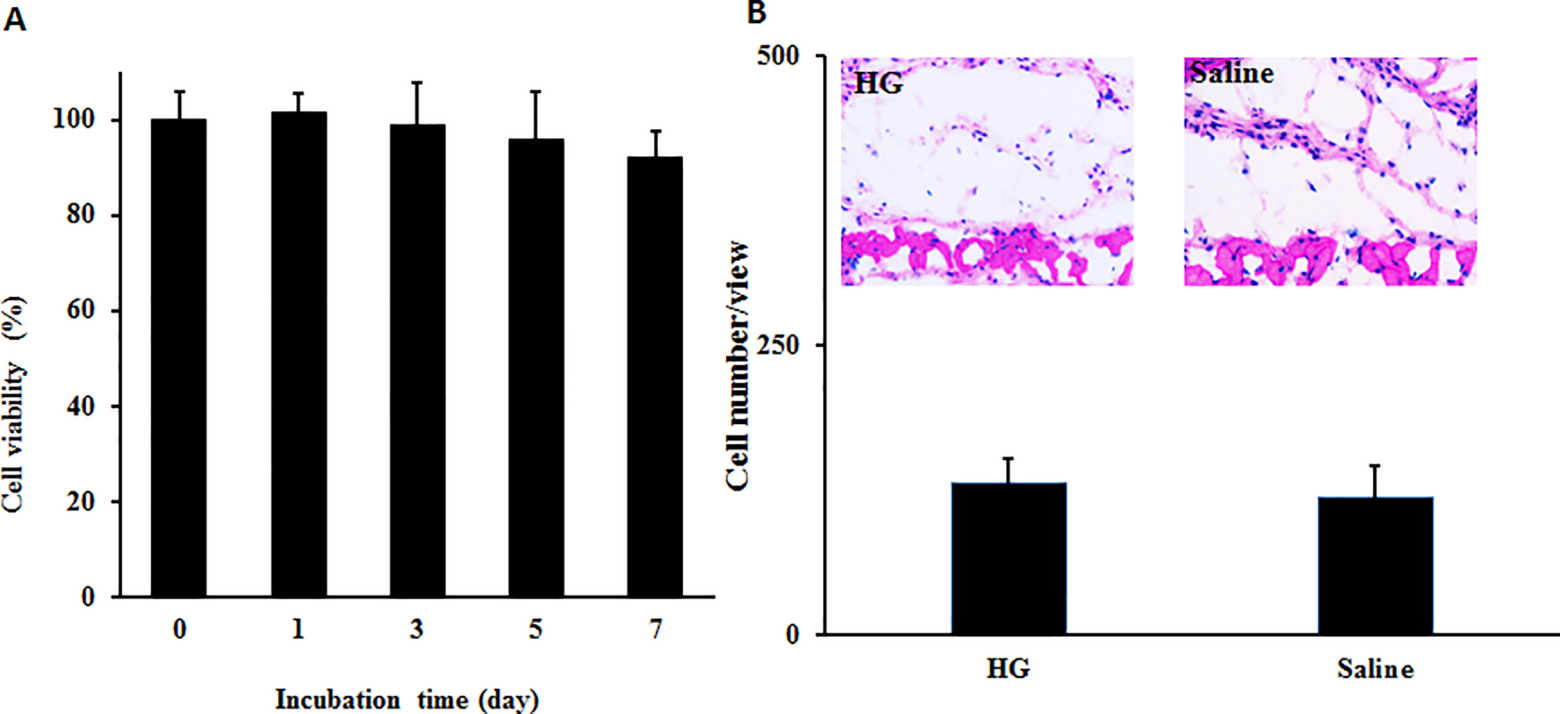
*In vitro* cell compatibility and *in vivo* tissue compatibility of HG was assessed. (A) HG was incubated with DMEM media for different periods of time (1, 3, 5 and 7 days) to generate conditioned media. The cell compatibility of the conditioned media was then evaluated using 3T3 fibroblasts and MTT assay. (B) Balb/c mice were subcutaneously implanted with HG or saline as the control. After implantation for 7 days, the animals were sacrificed and the implant-surrounding tissues were recovered for histological analyses. H&E stain and implant-associated cell numbers support that HG implant exerted minimal tissue response similar to saline control.

Next, we determined cell toxicity of the HG using conditioned media incubated with hydrogel for 1, 3, 5, and 7 days. As expected, HG-conditioned media did not alter cell survival (Fig 2A). These results suggest that there is no leachable cell toxin in the HG. Using a mouse subcutaneous implantation and saline as the control, biocompatibility of the polymer was evaluated in vivo. By counting the cells at the interface between cells and implants, we found that there was no statistical significance between cells recruited at HG implant sites vs. saline control (Fig 2B). These results imply that the HG has good tissue compatibility and is in agreement with earlier work confirming the excellent compatibility properties of polyethylene glycol-based hydrogel analogues [18, 32].

**Fig 3:**
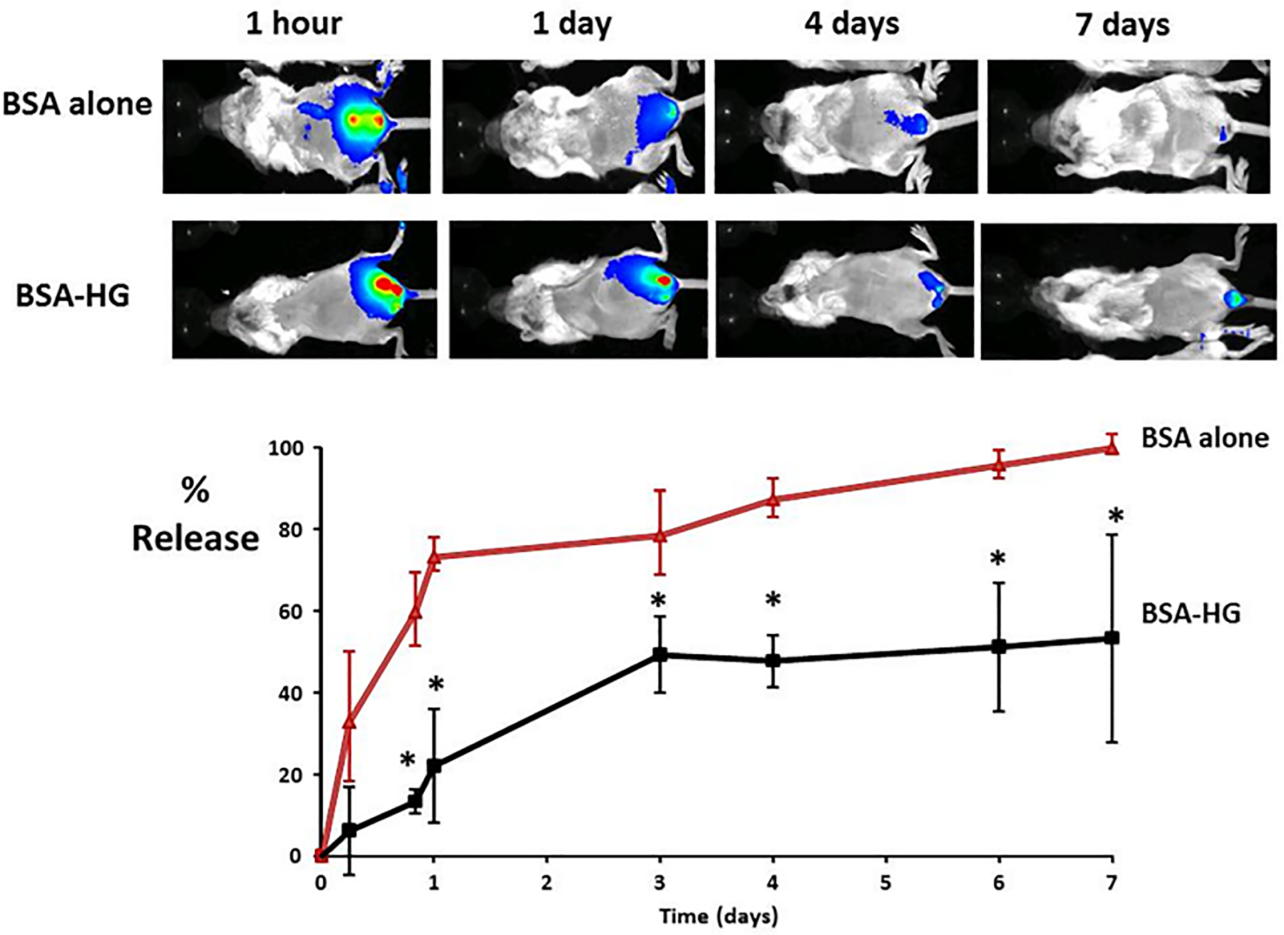
Release kinetics from PEG hydrogels. Upper panels are representative images of BSA-NIR fluorescence intensities at intravaginal injections as a function of time. Release kinetics from hydrogels (BSA-HG) are compared with that of BSA without HG. The percent BSA released was calculated based on the following formula. Release % = 100X [(Fluorescent intensity at time 0)–(fluorescent intensity at time T)] / (Fluorescent intensity at time 0). Note that release rates are delayed with HG compared with BSA alone. *P < 0.05.

The ability of the HG to release protein into the matrix of the vaginal wall was evaluated using bovine serum albumin (BSA) with a similar molecular mass as fibulin-5 as a model protein [33, 34]. NIR-labeled BSA was injected into the vaginal wall of mice with or without the presence of HGs (100μl, 0.1mg/ml BSA, 25 wt % HG). Release of BSA-NIR was then monitored daily using Kodak In Vivo FX Pro system. We found that the hydrogel exhibited good protein slow release properties *in vivo*. Seven days after administration, almost 100% BSA diffused out in absence of HG. With HG, however, approximately 50% BSA remained in the vaginal wall (Fig 3B). These results lend strong support to the use of such hydrogel platforms to deliver a wide variety of drugs and proteins for treating a wide variety of vaginal pathology.

### Fibulin-5 hydrogels inhibit vaginal MMP-9 enzyme activity

**Fig 4:**
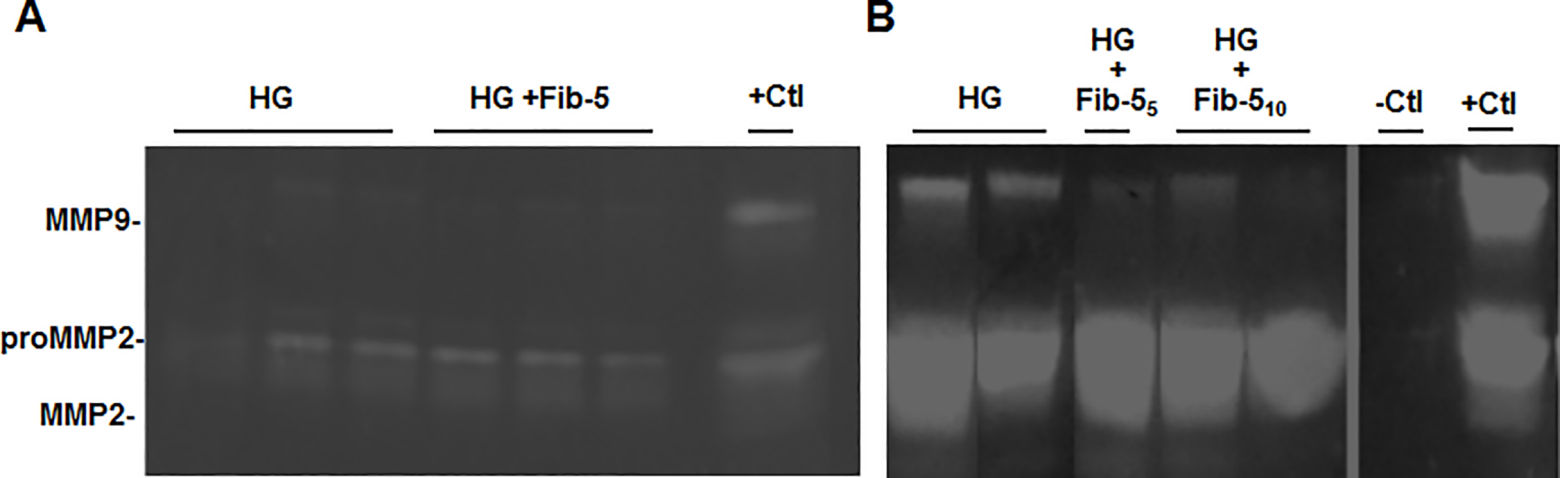
**Effect of HG ± fibulin-5 on MMP-9 activity in vaginal stromal cells from WT (A) or Fbln5**^**-/-**^ **(B) mice. HG,** hydrogel; **Fib-5**_**5,**_ recombinant fibulin-5 5 μg/ml; **Fib-5**_**10,**_ recombinant fibulin-5 10 μg/ml. **-Ctl,** vaginal tissues from MMP-9 KO mice; **+Ctl,** vaginal tissues from Fbln5^-/-^ mice.

Since fibulin-5 is absent in Fbln5^-/-^ mice, we used these animals to test incorporation of recombinant fibulin-5 in the vaginal wall. Recombinant fibulin-5 was generated with a His-tag and was recognized as a nonglycosylated protein of 51 kDa. Matrix fibulin-5, on the other hand, is glycosylated and migrates at 60 kDa. Previously, we showed that fibulin-5 inhibits vaginal MMP-9 through its RGD domain that binds to integrins and blocks fibronectin-induced upregulation of MMP-9 [6]. In this study, we used confluent stromal cells from Fbln5^-/-^ mice in which endogenous MMP-9 was activated. Baseline MMP-9 was not as active in preconfluent cells used previously and required fibronectin for activation. Here, MMP9 induction was apparent in confluent cells suggesting that confluency activates integrins in these cells and in the absence of fibulin-5 leads to induction of MMP9. To determine if HG was biocompatible and bioactive, vaginal stromal cells from wild type (WT) or *Fbln5*^*-/-*^ mice were treated with either HG alone or HG+fibulin-5. Hydrogel (ice cold 50 μl) was applied to the media of confluent cells where it gelled within seconds. Cells were examined microscopically and conditioned media analyzed for MMP2 and MMP9 activity using gelatin zymography (Fig 4). Cells appeared viable with no change in morphology and no dead floating cells with HG treatment. MMP-9 was absent in confluent WT stromal cells treated with HG±fibulin-5 (Fig 4A). In contrast, baseline MMP-9 was increased in *Fbln5*^*-/-*^ cells compared with WT. High basal MMP-9 activity was not altered by HG alone, but decreased when combined with fibulin-5 at 5 or 10 μg/ml (Fig 4B).

### Fbln5 knockout mice as a model system to study delivery of fibulin-5

**Fig 5:**
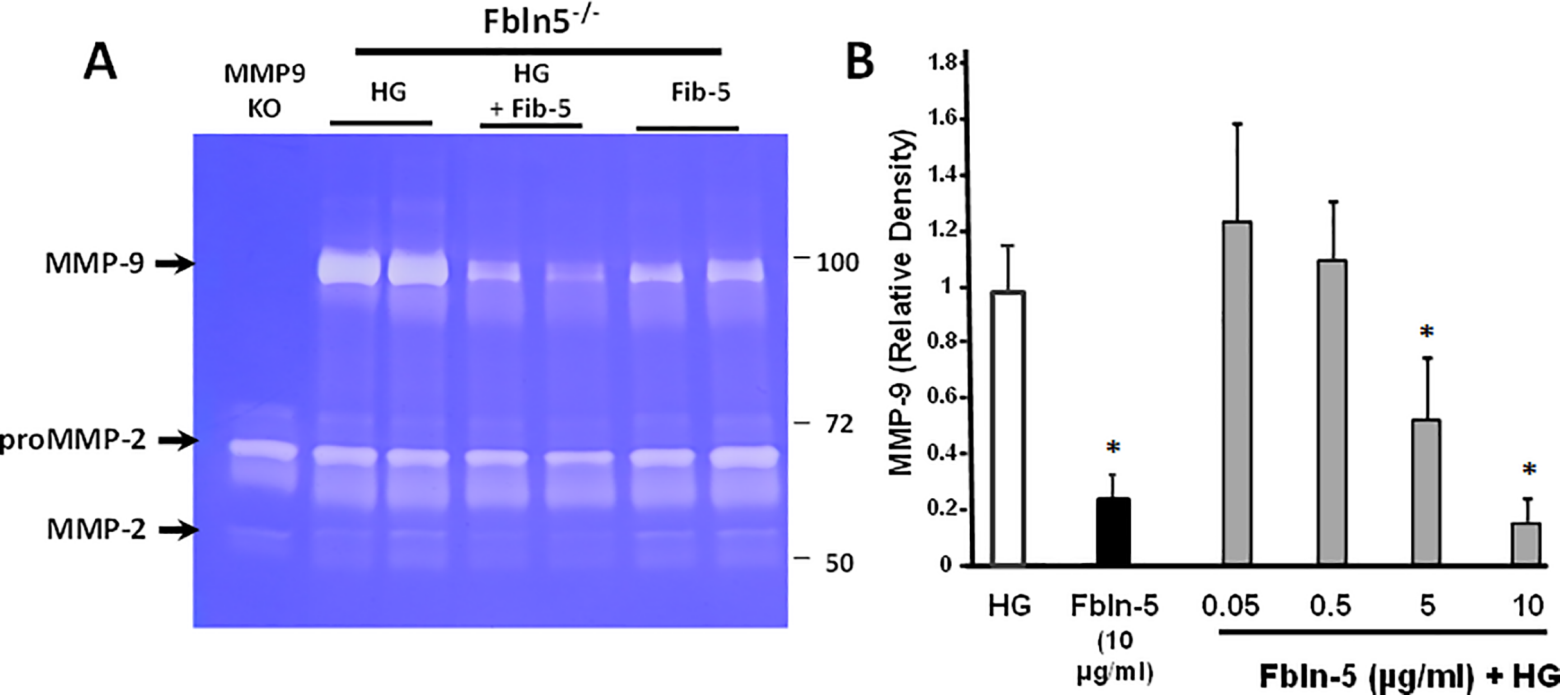
Effect of HG ± fibulin-5 on vaginal MMP-9 and MMP-2 in *Fbln5* KO mice treated for 7 d. **A.** Gelatin zymography with protein extracts (10 μg/lane) from adult *Fbln5*^*-/-*^ mice injected with hydrogel alone (HG), HG incorporated with fibulin-5 (5 ug/ml), or fibulin-5 alone x 7 d. *Mmp9* KO was used as a neg ctl. **B.** Cumulative results of 34 mice treated with HG ± various doses of fibulin-5 x 7 d. *P < 0.05 compared with HG alone, ANOVA.

To test the concept that implantation of HGs containing recombinant fibulin-5 may rescue protease activation in the vaginal wall of *Fbln5*^*-/-*^ mice, 34 KO animals without prolapse were injected with thermosensitive HGs ± fibulin-5 (0.05–10 ug/ml). After one week, tissues were harvested and analyzed for MMP-2 and MMP-9 using quantitative zymography (Fig 5). Since pro- and active forms of MMP9 are difficult to separate using zymography, data are expressed as total MMP9. Compared with hydrogel alone, MMP-9 activity was inhibited significantly with 5 ug/ml and dramatically at 10 ug/ml indicating that recombinant fibulin-5 remained bioactive for at least 7 d.

In vivo without injury, fibulin-5 inhibited MMP-9 activity for up to 4 weeks after injection. Although stable and incorporated in the vaginal matrix for 7 d, immunoreactive fibulin-5 was not detected in most animals after 2 weeks. These findings are compatible with the idea that the RGD domain of fibulin-5 remains bound to integrins and thereby blocks MMP9 activation, but the majority of the protein is degraded by endogenous proteases. It is also possible that MMP-9 remains suppressed unless activated by injury, stretch, or cellular stress.

**Fig 6:**
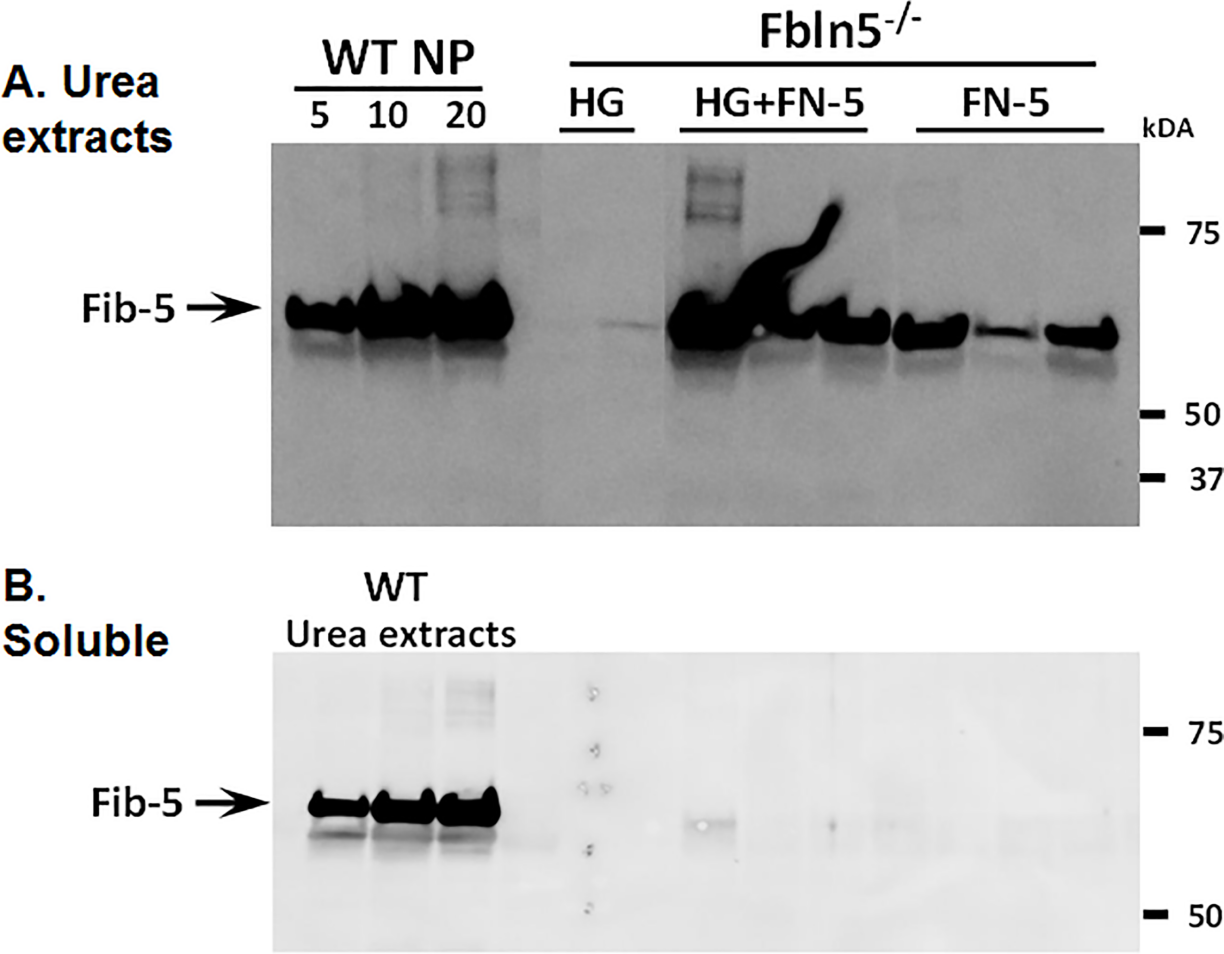
Recovery of fibulin-5 in vaginal tissues from *Fbln5*^*-/-*^ mice injected with hydrogel (HG) ± recombinant fibulin-5 (FN5). Urea extracts (10 μg/lane, **A**) revealed incorporation of fibulin-5 in matrix fractions of KO animals 7 d after injection with HG+FN-5 (equivalent to that in WT animals). WT urea extracts were used as the + control in the soluble fractions (**B**).

Next, we sought to determine if intact fibulin-5 was incorporated in the vaginal matrix after one week (Fig 6). Urea-extracted vaginal matrix was used as a positive control. As expected, fibulin-5 was not expressed in the vaginal wall of KO animals treated with hydrogel alone. However, fibulin-5 was recovered from the urea-extracted matrix of animals treated with hydrogel + fibulin-5 (Fig 6). Interestingly, immunoreactive fibulin-5 was absent in soluble protein fractions of the vaginal wall. Hence, these results provide compelling evidence that (i) fibulin-5-incorporated hydrogels are bioactive, and (ii) fibulin-5 replacement becomes incorporated in the urea-extractable matrix.

**Fig 7:**
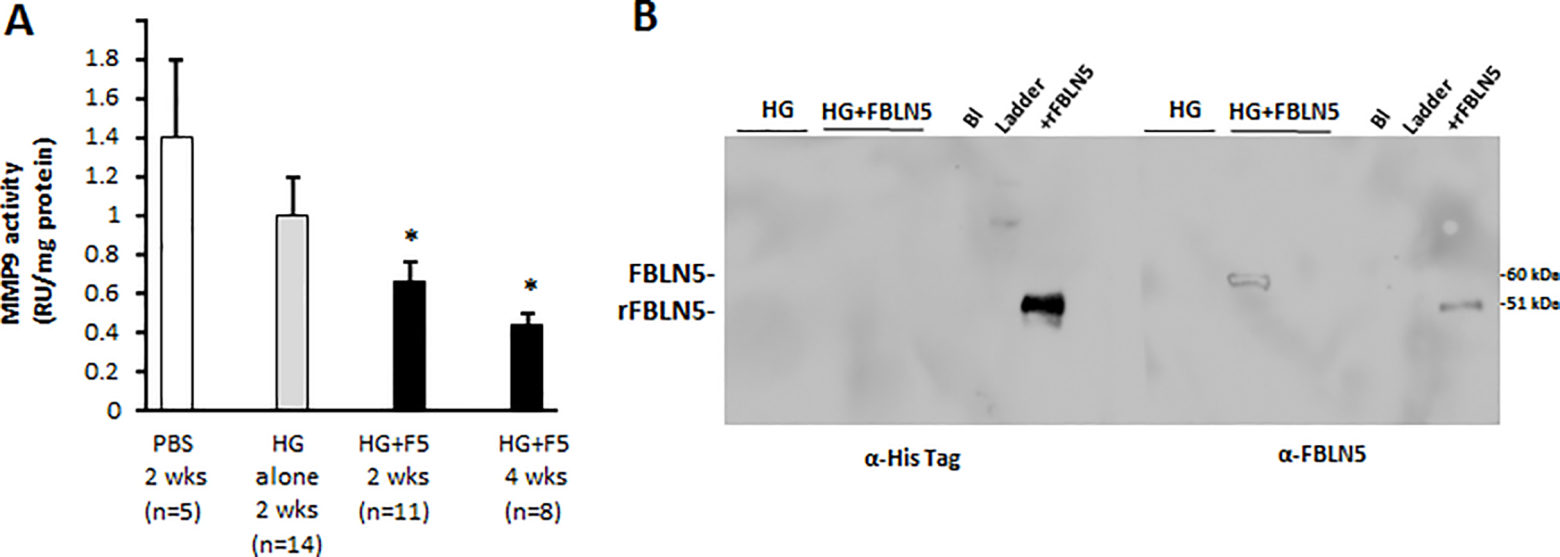
Long-term effects of HG ± fibulin-5 on vaginal MMP-9 in *Fbln5* KO mice treated for 14–28 d. **A.** Cumulative results of MMP-9 activity in vaginal tissues from 38 mice treated with HG ± fibulin-5 (10 μg/ml) for 2–4 weeks. *P < 0.05 compared with HG alone, ANOVA. **B.** Immunoblot analysis of urea-extracted protein from vaginal tissues of Fbln5^-/-^ mice treated with HG or HG + FBLN5 (10 μg/ml) x 2 weeks. The left blot was incubated with **anti- His Tag** antibody whereas the right blot was incubated with anti-fibulin-5. Positive controls on each blot are recombinant fibulin-5 (rFBLN5, 200 ng). **Bl**, blank.

Experiments were then conducted to determine the longevity of the effects of fibulin-5 on the vaginal wall. Specifically, *Fbln5*^*-/-*^ nulliparous mice were injected with PBS, HG alone, or HG+fibulin-5 and tissues harvested 2 and 4 weeks after treatment. Fibulin-5+HG, but not HG alone, resulted in significant inhibition of baseline vaginal MMP9 activity up to 4 weeks by up to ~50% (Fig 7A). MMP-2, on the other hand, was not affected. Immunoblotting was used to determine if recombinant fibulin-5 could be recovered from the vaginal wall 2 weeks after injection. Vaginal tissues from 2 animals were pooled to obtain enough urea-extracted protein for analysis. Using an antibody that recognizes the His-tag of recombinant fibulin-5, but not endogenous protein, we found that recombinant fibulin-5 was not recovered in either soluble or urea-extracted fractions of the injected vaginal wall (Fig 7B). Interestingly, however, fibulin-5 was incorporated in one of 5 animals injected with HG+fibulin-5. The molecular weight of the protein was that of endogenous glycosylated vaginal protein. Nonetheless, it is noteworthy that MMP9 remained suppressed despite lack of incorporation of fibulin-5 in the majority of animals.

**Fig 8:**
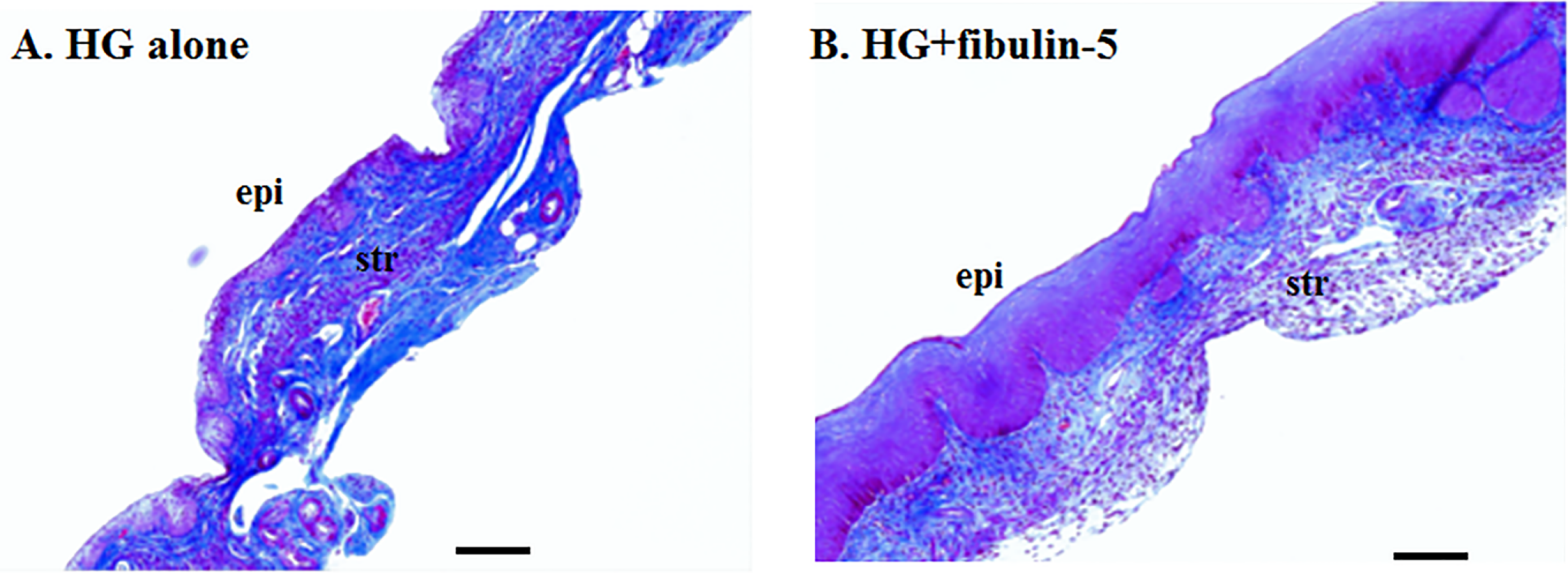
Trichrome-stained sections of the vaginal wall of *Fbln5*^*-/-*^ mice treated with HG (A) or HG+fibulin-5 (10 μg/ml). Note increased thickness of epithelial and stromal layers in animals treated with fibulin-5. Bar = 20 μm.

To determine if fibulin-5-induced inhibition of vaginal MMP-9 was associated with histomorphologic change in the vaginal wall of Fbln5^-/-^ mice, Masson’s trichrome stained cross-sections of injected vaginal wall were analyzed by examiners blinded to treatment (Fig 8). Histomorphology revealed that HG+fibulin-5 resulted in significant increases in vaginal epithelial (164 ± 77.0 compared with 66 ± 17.1 μm, *p =* 0.05) and stromal thickness (231 ± 68.5 versus 175 ± 39.7 μm, *p* = 0.16), suggesting that inhibition of MMP-9 activity was associated with increased thickness of the vaginal wall.

It is somewhat paradoxical to find that fibulin-5-induced inhibition of vaginal MMP-9 was associated with significant increases in vaginal epithelial thickness. It is well known that vaginal mesenchyme induces epithelial differentiation. For example, skin grafts used for neovagina formation in women with vaginal agenesis develop vaginal mucosal epithelium[35]. We suggest, therefore, that stromal fibulin-5 inhibits MMP-9 protease activity which alters epithelial differentiation via improved mesenchymal-derived inductive capacity. Further experiments are necessary to test this hypothesis.

Overall, these results indicate that slow release of purified fibulin-5 or other matrix proteins may prevent or abrogate vaginal proteases and thereby promote matrix synthesis in the vaginal wall.
